# Long term effect of a school based intervention to prevent chronic diseases in Tunisia, 2009–2015

**DOI:** 10.4314/ahs.v17i4.23

**Published:** 2017-12

**Authors:** Rim Ghammam, Jihen Maatoug, Nawel Zammit, Raoudha Kebaili, Lamia Boughammoura, Mustafa Al'Absi, Harry Lando, Hassen Ghannem

**Affiliations:** 1 Department of Epidemiology, University Hospital Farhat Hached, Sousse Tunisia; 2 Department of Pediatrics, University Hospital Farhat Hached , Sousse Tunisia; 3 Duluth Medical Research Institute, University of Minnesota, USA; 4 Department of epidemiology & Community Health University of Minnesota, USA

**Keywords:** Schools, lifestyle, intervention

## Abstract

**Background & Objectives:**

We aimed to evaluate the long term effect of school based intervention to prevent non- communicable disease risk factors.

**Methods:**

It was a quasi experimental study conducted during the period of 2009–2015. We involved school children aged from 11 to 16 years old. For the assessment of the program's effectiveness, subjects in both groups were examined at baseline, at the end of the 3-year intervention period and at the follow-up, one year after program's cessation.

**Results:**

In the intervention group, the prevalence of school children who reported to be eating 5 fruits and vegetable sdaily increased significantly from 30.0% at pre-assessment to 33.2% at post-assessment, one year after (p=0.02, p=0.41 respectively). For the control group, this prevalence had significantly decreased from 40.2% at baseline to 35.0% at post-intervention, at the follow up, this proportion increased to 44.5%(p=0.001, p<10^−3^ respectively). Concerning smoking habits, we observed a decreasing trend in the intervention group from 5.7% at pre-assessment, to 4.8% at post-assessment and to 3.4% at the follow-up (p=0.19 and p=0.25 respectively). There was also a significant decrease in school children who did recommended physical activity in the same group.

**Conclusion:**

The present work showed that interventions promoting healthy lifestyles should be maintained. Developing countries should be encouraged and supported to design, conduct, and evaluate robust preventive interventions.

## Introduction

With non-communicable disease (NCDs) conditions accounting for nearly two-thirds of deaths worldwide, the emergence of chronic diseases as the predominant challenge to global health is undisputed^1^. NCDs are one of the major health and development challenges of the 21^st^ century^2^. As the leading cause of death globally, NCDs were responsible for 38 million (68%) of the world's 56 million deaths in 2012. More than 40% of them (16 million) were premature deaths under age 70 years. Almost three quarters of all NCDs deaths (28 million), and the majority of premature deaths (82%), occur in low and middle-income countries (LMIC)^2^. Unhealthy dietary behaviors, physical inactivity, and tobacco use contribute to NCDs and other health conditions, including obesity, diabetes and asthma^3^. These behaviors often established during childhood and adolescence, extend into adulthood, are interrelated, and preventable^3^.

The number of adolescents who are overweight or obese is increasing in both low- and high-income countries. Available survey data indicates that less than 1 in every 4 adolescents meets the recommended guidelines for physical activity. Developing healthy eating and exercise habits at this age are foundations for good health in adulthood. Reducing the marketing of foods high in saturated fats, trans-fatty acids, free sugars, or salt and providing access to healthy foods and opportunities to engage in physical activity are important for all but especially children and adolescents. The vast majority of people using tobacco today began doing so when they were adolescents. Globally, at least 1 in 10 younger adolescents (aged 13 to 15) uses tobacco, although there are areas where this figure is much higher. Cigarette smoking seems to be decreasing among younger adolescents in some high-income countries^4^.

Tunisia as most LMIC is facing an epidemiological transition with increase of NCDs (5–7). So, promoting healthy practices during adolescence, and taking steps to better protect young people from health risks are critical for the prevention of health problems in adulthood, and for countries' future health and social infrastructure^4^.

The “Together in Health” project for the prevention of risk factors for NCDs is an example of a loco-regional initiative. It was implemented by the Chronic Disease Prevention Research Center (CDPRC) in Sousse Tunisia with the partnership of different national and international partners^8^. This initiative has shown that it is possible to design and implement activities to promote health in various settings with the support of the community^9^. The majority of studies did not provide data on longterm follow-up, so the sustainability of this approach is largely unknown^10^. Additionally, most studies come from high-income contexts, mostly from North America. Only few studies were conducted outside of high-income countries, and especially in low-income countries^10^. To the best of our knowledge, there is no study conducted in Tunisia examining the long term effect of a community based intervention to promote healthy life style. The aim of the current study was to evaluate the long term effect of recent school based intervention to prevent NCDs risk factors: project “Together in Health” in Tunisia

## Methods

### Study design

**Figure 1:**
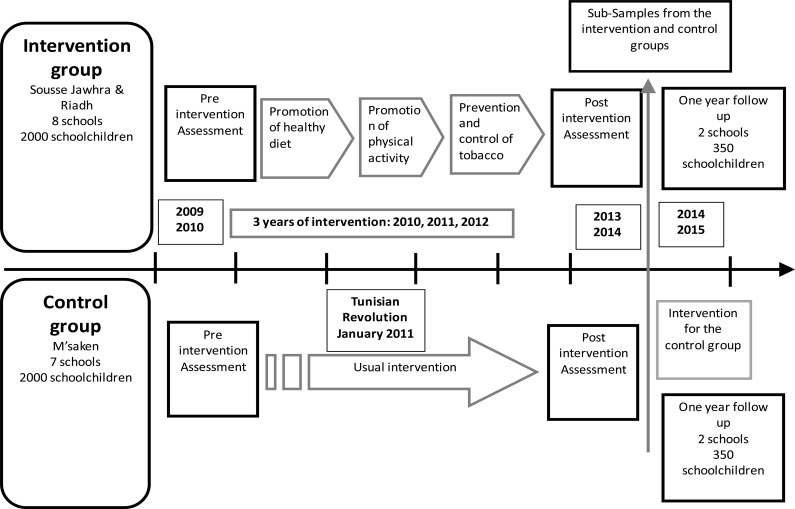
Study design of three year community based intervention for chronic disease prevention in Sousse Tunisia with one year Follow-up

The study was a quasi-experimental study with intervention and control groups. The intervention group was located in the delegation of Sousse Jawhra and Sousse Erriadh. The control group was located in delegation of Msaken. For the assessment of the program's effectiveness, subjects in the intervention and control groups were examined at baseline, at the end of the 3-year intervention period and at a follow-up, one year after program's cessation (Figure 1).

### Studied population

The study involved school children of colleges aged 11 to 16 years in 7^th^ and 9^th^ grade. The intervention and the control groups were randomly selected from all colleges of respectively intervention and control zone. So there were nine intervention schools and eight control schools. The one year follow up constituted only four schools, two from the intervention group and two from the control group as representative of each delegation. The 3 measurements (baseline, 1^st^ and 2^nd^ evaluation) were performed on independent groups.

For the follow up analysis, schools were selected according to the number of school children, their location and their accessibility.

### Data collection

A standardized, pre-tested questionnaire in Arabic was used to evaluate knowledge of, attitudes towards and beliefs on the three risk factors of chronic disease: unhealthy diet, physical inactivity and tobacco use. The questionnaire was self administered in classes. Trained medical doctors assisted children in filling the questionnaire. We also collected biometric measures such as height and weight. Body weight was recorded to the nearest 0.1kg using a portable electronic scale. Standing height was measured with the participants in bare feet to the nearest 0.5 cm. Waist circumference was measured to the nearest 0.1cm using a non-stretchable standard tape measure. The measurement was taken over a light clothing, at the smallest diameter between the costal margin and the iliac crest.

### Variable definition

We defined the recommended amount of fruits and vegetables as 5 or more servings per day^11^.

The recommended level of physical activity for children was established according to the definitions by the World Health Organization^12^. Sedentarily behavior was defined as any seated activity for more than 2 hours per day.

Body mass index was computed as the ratio of the body weight to the body height squared and expressed as kg/m^2^. To define overweight and obesity, we used recent international cut-off values of BMI according to age and sex^13^.

Smoker was defined as the person who smoked at least one cigarette in the last month^14^. Daily smoker is the person who smokes at least one cigarette per day.

### Intervention program

The three-year intervention program required several preparations before it starts. A multidisciplinary team has worked on the intervention program. Information and training meetings for teachers, inspectors of the life sciences and earth, physical activity were organized. In each intervention college, student leader groups were organized and trained in order to play the role of peers in the fight against unhealthy lifestyles. Thereafter, these leaders organized an awareness day for prevention of tobacco smoking in their colleges. The project team distributed leaflets to students for support of the intervention. A Face-book group called “Together for Health” was created to allow the exchange of information between students on the topic. The project team used this group to upload photos and videos of the different activities which took place in schools with the collaboration of the CDPRC^15^.

For ethical considerations, a delayed intervention was performed for the control group after post-assessment data collection in both groups. The delayed intervention consisted on the distribution of the same flyers used in the intervention group with educational messages about smoking, physical activity and healthy eating; the organization of open days with a screening of NCDs risk factors; the distribution of sports equipment for schools and the diffusion of educational message through thefacebook group “Together for Health”.

### Data analysis

Continuous variables were presented as Mean (SD) and categorical ones as percentages (%). All statistical analyses were carried out by using the SPSS 10.0 software for Windows. We used Chi square test to compare percentages and independent sample t test to compare means between groups. The level of statistical significance was set at p<0.05 level.

### Ethical consideration

This study was undertaken with respect of the rights and the integrity of people. Ethical clearance was obtained from the Ethical Committee of Farhat Hached University Hospital in Sousse, Tunisia. Parents and children gave their consent and they were able to refuse participation. We used an anonymous questionnaire that did not contain the name or the address of students. The intervention consisted of educational messages which didnot have any harmful consequences for school children.

## Results

The characteristics of the studied population from baseline to one year follow-up are presented in Table 1.

**Table 1:** Socio-demographic characteristics of school children participating in the study in the Region of Sousse, Tunisia, 2009–2015.

	Intervention group				Control group					
	
	Pre-assessment	Post-assessment	Follow up	P value		Pre-assessment	Post-assessment	Follow up	P value	
				pre-post	post-follow up				pre-post	post-follow up
Response rate n(%)	1929(93.1)	2170(91.9)	370(90,02)	-	-	2074(96.0)	2105(93.9)	381(87,18)	-	-
Age										
,14 years n(%)	1074(55.7)	1178(54.7)	212(57.5)	**0.516**	**0.320**	970(46.8)	1107(52.7)	180(47.7)	**<10^−3^**	**0.073**
≥14 years n(%)	855(44.3)	977(45.3)	157(42.5)			1104(53.2)	993(47.3)	197(52.3)		
Gender										
Girls n(%)	961(49.8)	1100(51.3)	207(56.9)	**0.359**	**0.048**	1109(53.5)	1100(52.3)	184(49.3)	**0.460**	**0.285**
Boys n(%)	968(50.2)	1046(48.7)	157(43.1)			965(46.5)	1002(47.7)	189(50.7)		

In the intervention group, the prevalence of school children who reported to be eating 5 fruits and vegetables daily increased significantly from 30.0% at pre-assessment to 33.2 at post assessment (p=0.02) and decreased one year after to 31% (p=0.41). Among boys, this proportion was 33.3%, 37.2% and 28.8% at pre-intervention, post-intervention and follow up respectively (p=0.07, p=0.04). For the control group, this prevalence significantly decreased from 40.2% at baseline to 35.0% at post-intervention (p=0.001) and at the follow up, this proportion increased to 44.5% (p<10-3). Among boys, this prevalence was 44.0%, 37.1% and 39.8% at pre-intervention, post-intervention and follow up respectively (p=0.002, p=0.5). (table 2)

**Table 2:** Evaluation of eating habits among children participating in the study in the Region of Sousse, Tunisia, 2009–2015.

	Intervention group					Control group				
	
	Pre-assessment	Post-assessment	Follow up	P value		Pre-assessment	Post-assessment	Follow up	P value	
				pre-post	post-follow up				pre-post	post- follow up
5 fruit and vegetable daily	565(30.0)	703(33.2)	109(31.0)	**0.026**	**0.410**	821(40.2)	694(35.0)	158(44.5)	**0.001**	**<10^−3^**
Boys n(%)	314(33.3)	381(37.2)	42(28.8)	**0.070**	**0.047**	419(44.0)	352(37.1)	72(39.8)	**0.002**	**0.500**
Girls n(%)	251(26.6)	320(30.0)	64(32.0)	**0.097**	**0.566**	402(36.9)	340(32.9)	85(48.8)	**0.055**	**<10^−3^**
<14 years n(%)	319(30.2)	408(35.4)	66(31.7)	**0.009**	**0.304**	379(39.5)	401(37.9)	73(42.7)	**0.456**	**0.232**
≥14 years n(%)	246(29.7)	291(30.7)	42(29.4)	**0.640**	**0.748**	442(41.2)	304(32.6)	83(46.1)	**<10^−3^**	**<10^−3^**
Eat fast food 4 days/ week or more	358(18.7)	384(17.9)	53(14.9)	0.517	0.170	224(10.8)	322(15.9)	53(14.1)	**<10^−3^**	**0.369**

Concerning smoking habits among school children, the proportion of smokers was 5.7%, 4.8% and 3.4% respectively at pre-assessment, post-assessment and follow-up in the intervention group (p=0.19, p=0.25). This proportion was 7.5%, 9.2% and 6.4% at pre-assessment, post-assessment and follow-up in the control group respectively (p=0.04, p=0.07). In the control group, the proportion of smokers increased significantly among girls and under-14-year old children (p<10-3, p=0.006). This increase wasn't significant among boys and older-than-14 year old children (p=0.4, p=0.24). One year after, there was a significant decrease only among girls (p=0.005) in the control group. In the intervention group, the increase of smoker's proportion wasn't significant in relation to age and sex (table 3).

**Table 3:** Evaluation of smoking habits among children participating in the study in the Region of Sousse, Tunisia, 2009–2015.

	Intervention group					Control group				
	
	Pre-assessment	Post-assessment	Follow up	P value		Pre-assessment	Post-assessment	Follow up	P value	
				pre-post	post-follow up				pre-post	post- follow up
Smokers n(%)	110(5.7)	73(4.8)	12(3.4)	**0.191**	**0.254**	155(7.5)	193(9.2)	24(6.4)	**0.047**	**0.078**
Boys n(%)	85(8.8)	81(7.7)	8(5.4)	**0.398**	**0.310**	143(14.8)	136(13.6)	22(11.9)	**0.429**	**0.536**
Girls n(%)	25(2.6)	23(2.1)	2(1.0)	**0.443**	**0.465**	12(1.1)	57(5.2)	1(0.6)	**<10^−3^**	**0.005**
<14 years n(%)	37(3.4)	41(3.5)	4(2.0)	**0.963**	**0.254**	32(3.3)	65(5.9)	11(6.3)	**0.006**	**0.843**
≥14 years n(%)	73(8.5)	63(6.4)	8(5.5)	**0.089**	**0.667**	123(11.1)	127(12.8)	13(6.7)	**0.245**	**0.017**
Daly smokers n(%)	39(2.0)	29(1.4)	7(2.1)	**0.107**	**0.386**	73(3.5)	51(2.6)	12(3.4)	**0.088**	**0.503**
Age of first cigarette year(Sd)	10.37 (3.01)	10.74 (3.10)	11.84 (2.14)	**0.179**	**<10^−3^**	10.77 (3.21)	11.11 (2.94)	10.82 (3.20)	**0.141**	**0.092**

There was a significant decrease (p=0.01, p=0.001 respectively) in school children who did recommended physical activity in the intervention group, dropping from 29.1% in the pre-intervention to 25.5% in the post-intervention to 17.8% in the follow up. At post assessment, this decrease was significant among boys and over-14-year old school children. One year after, this proportion decreased significantly among girls and under-14-year old pupils. In the control group, the proportion of schoolchildren who did recommended physical activity increased from 21.1% in the pre-intervention to 21.2% in the post-intervention to 26.5% in the follow up, but the difference was only significant in the follow up (p=0.91, p=0.02 respectively). By the end of the intervention program, there was no significant difference with regard to age or gender. One year after, there was been a significant increase only among boys (p=0.01) (table 4)

**Table 4:** evaluation of physical activity and weight excess among children participating in the study in the Region of Sousse, Tunisia, 2009–2015

	Intervention group					Control group				
	Pre-assessment	Post-assessment	Follow up	P value		Pre-assessment	Post-assessment	Follow up	P value	
				pre-post	post-follow up				pre-post	post-follow up
Recommended physical activity n (%)	554(29.1)	537(25.5)	63(17.8)	**0.010**	**0.001**	434(21.1)	425(21.2)	99(26.5)	**0.912**	**0.023**
Boys n(%)	431(44.9)	407(39.8)	50(33.1)	**0.021**	**0.071**	355(37.1)	342(35.8)	85(45.7)	**0.560**	**0.012**
Girls n (% )	123(13.0)	126(11.9)	12(6.1)	**0.439**	**0.030**	79(7.2)	82(7.9)	12(6.7)	**0.530**	**0.500**
< 14 years n(%)	320(30.1)	326(28.5)	35(16.8)	**0.392**	**0.003**	203(21.1)	229(21.5)	49(27.8)	**0.817**	**0.062**
≥14 years n(%)	234(27.8)	207(21.9)	27(18.6)	**0.004**	**0.145**	231(21.0)	194(20.8)	49(25.3)	**0.920**	**0.173**
Weekday screen time>2h per day n(%)	700(36.9)	809(38.3)	119(34.4)	**0.365**	**0.166**	699(33.9)	635(31.8)	163(43.6)	**0.161**	**<10^−3^**
Sunday screen time> 2h n(%)	1171(62.9)	1279(62.8)	200(58.1)	**0.950**	**0.100**	1280(64.0)	1113(57.0)	246(67.0)	**<10^−3^**	**<10^−3^**
Norma l weight n (%)	1396(72.4)	1606(75.5)	263(72.9)	**0.024**	**0.283**	1658(80.0)	1565(76.9)	287(77.4)	**0.016**	**0.844**
Overweight n (%)	396(20.6)	382(18.0)	70(19.4)	**0.036**	**0.531**	321 (15.5)	328(16.1)	56(15.1)	**0.602**	**0.634**
Obesity n (%)	135(7.0)	138(6.5)	28(7.8)	**0.513**	**0.373**	94(4.5)	141(6.9)	28(7.5)	**<10^−3^**	**0.644**
Overweight and obesity n(%)	533(27.6)	564(26.0)	98(27.1)	**0.236**	**0.643**	416(20.1)	540(25.7)	84(22.6)	**<10^−3^**	**0.211**
< 14 years n(%)	319(29.7)	318(27.0)	64(30.2)	**0.154**	**0.337**	214(22.1)	281(25.4)	37(20.6)	**0.076**	**0.163**
≥14 years n(%)	214(25.0)	231(23.6)	34(22.8)	**0.490**	**0.825**	202(18.3)	254(25.6)	47(24.6)	**<10^−3^**	**0.777**
Boys n(%)	261(27.0)	247(23.6)	31(20.0)	**0.084**	**0.319**	193(20.0)	247(24.7)	42(22.5)	**0.013**	**0.521**
Girls n (% )	272(28.3)	292(26.6)	65(32.3)	**0.397**	**0.096**	223(20.1)	290(26.4)	40(22.1)	**<10^−3^**	**0.224**

In the intervention group, the proportion of overweight and obese school children was 27.6%, 26.0% and 27.1% respectively at baseline, post-intervention and follow-up (p=0.23, p=0.64 respectively). There wasn't a significant difference concerning sex or age in overweight and obesity within three measurements. In the control group, the proportion of overweight and obesity was 20.1%, 25.7% and 22.6% respectively at baseline, post-intervention and follow-up (p<10-3, p= 0.21 respectively). With the end of the intervention program, this proportion significantly rose among girls (<10-3), boys (p=0.01) and children aged-over-14-years. One year after, the proportion of overweight and obesity by sex and age didn't change significantly (table 4).

## Discussion

The baseline results indicate an important proportion of low physical activity, tobacco use, overweight and obesity. The effectiveness of the three years' intervention program in students of Sousse Tunisia has been evaluated in previous studies^15^–^18^. Tracking the effects of the intervention in schools a year after its achievement has shown that it still has some positive effects. In fact, we have a stability of the positive effect in recommended serving of fruits and vegetables (30%, 33.2% and 31% respectively in the 3 times of measurement), (p= 0.02 and 0.4) particularly among girls and children under 14 years of age. The same positive effect was observed on smoking habit. Our program also kept a benefic effect on obesity and overweight especially among boys (27%, 23% and 20% respectively in the 3 times of measurement) and older than 14 years old school children (25%, 23.6% and 22.8% respectively in the 3 times of measurement). For recommended physical activity, the intervention didn't have a positive effect. In the control group, we observed positive effects on physical activity, fruits and vegetables consumption one year after the end of the program. This effect may be explained by the short term effect of the differed intervention that was made for the control group before the follow-up evaluation.

At the end of 5 years, the school based intervention program had a positive effect on school children eating habits. the revues of Evans et al^19^ and Knai et al^20^ showed that multi-component programs that motivate and engage children and families to change their eating behaviors, as our program, tended to result in larger improvements in fruits and vegetables intake than single-component programs that provide and distribute free or subsidized fruit and vegetables. Very few studies collected follow-up data a full year after the intervention. The effect of the current study was reduced one year after the end of the program. These changes are consistent with other research findings in this area, and are important from a public health perspective^21^. Bere et al^22^ showed a moderate long-term impact on fruit and vegetable intake, which indicated that, if intervention programs are to have an impact on the health of children, the programs must run continuously over long periods of time , should not be considered as one-off solutions^19^,^21^,^23^, should be started at an early age and continued to adulthood^24^. Although it is impossible to identify the main variable(s) responsible for student behavior change, it is likely that the intermittent reinforcement component implemented at the point of performance played a major role in increasing students' consumption^21^,^23^.

In our study, there was significant decrease in physical activity in the intervention group in all three examinations. This may be explained by occurrence of the Tunisian Revolution in the middle of the project. So, environmental changes haven't taken place and there was a lack of security which represented a significant barrier to physical activity^10^. Increasing physical activity among children and adolescents is difficult as behavior is influenced by several factors including: personal factors; institutional, community, public policy, and the physical environment^25^. School-based physical activity interventions of longer duration may be needed to effect change in duration and rate of physical activity among grade school children. However, the evidence is less convincing for those attending secondary school^26^. Dobbins et al^27^ review showed that, school-based interventions that include some combination of school curricula, printed educational materials, seem to be more effective then educational sessions, physical activity specific sessions, and community-based initiatives. In order to produce sustainable effects, it may be necessary to widen the scope of the intervention to include the community so as to promote multiple environments that support active living as children move from childhood to adolescence to adulthood. Community-based strategies have been shown to be somewhat effective in promoting healthy behavior among populations^27^,^26^.

The intervention group had significant decrease on the prevalence of overweight children. However, the prevalence of overweight and obesity had significantly increased at the control group. The majority of program outcomes at least in the short term indicated change towards improvement, which supports continued action^28^. Few programs had follow-up exceeding a year and there is lack of programming to address the particular needs of sub-groups of children or gender specificities. Healthy eating, active living and mental well-being are the common elements of prevention programs for obesity and also for several chronic diseases associated with obesity. More attention needs to be paid to stakeholder involvement in program development and program integrity. Program design process should be developed to allow continual incorporation of new elements associated with greater program effectiveness such as type of project leader, nature of intervention delivery, etc.^28^

The finding of the current study revealed a moderate long term effect of the school based intervention on smoking behavior among school children. Some studies reported clear positive intervention effects^29^. In part, these effects were restricted to the culturally adapted intervention^30^. A review of long-term tobacco and drug use prevention intervention studies published since 1966 indicates that school- and community-based programs were effective in preventing or reducing adolescent cigarette, alcohol, and marijuana use across follow-up periods ranging from 2 to 15 years^31^. Langford et al demonstrated that both tobacco only and multiple risk behavior interventions are effective on reducing smoking in school children. The single Emotional well-being intervention gave an estimated effect in favor of the intervention^32^. Muller-Riemenschneider's review^33^ of behavioral interventions for smoking prevention provides moderate evidence for the long-term effectiveness of community-based and multisectorial interventions. For school-based interventions, however, the evidence of effectiveness was less convincing. Indeed, even in studies providing evidence of effectiveness, reductions in smoking rates were only modest. However, even current and comprehensive behavioral smoking prevention programs are only associated with a moderate reduction in smoking prevalence and they should be complemented by appropriate environmental strategies to achieve sustainable reductions in smoking rates on a broad population basis.^33^

The majority of studies did not provide data on longterm follow-up. So the sustainability of this approach is largely unknown. Studies were also often underpowered and relied heavily on self-reported data^32^.

## Limitations

First, there wasn't randomization between the two groups, that's why we are not able to confirm that all observed changes were due to the intervention program. Second, we used self-reported data to evaluate lifestyle risk factors. Self reported data is potentially subject to information bias^34^–^36^. Nevertheless, the possible subjectivity is the same at pre- and post-assessment and didn't affect the evolution due to the intervention. The follow-up was realized on sub-samples of the intervention and the control groups. This may be a limit when tracking the real effect of the intervention. Furthermore, it is possible that the control group was contaminated by the intervention effect since the two cities (Sousse and M'saken) are not very distant.

These limitations are off-set by several strengths. First, the training of investigators and the standardization of data collection. Second, The project “Together in health” was the first intervention at the community level in Tunisia through a quasi-experimental research design with a control group, with a relatively important duration of intervention and that took place with the occurrence of the Tunisian Revolution^10^. So, it was a challenge to make this project succeed and to assess its long-term effects.

## Conclusion

The present work showed that interventions promoting healthy life style should be maintained. A short intervention wasn't sufficient to reduce cardiovascular risk factors. Although evidence for the effectiveness of school-based interventions was inconclusive, evidence for the effectiveness of community-based and multisectorial interventions was somewhat stronger. More research is needed to examine in more depth, and for longer follow-up periods, the effectiveness of interventions promoting healthy lifestyles. Developing countries should be encouraged and supported to design, conduct, and evaluate robust preventive interventions. Evaluation should be included as part of the project plan.
